# Evolution of Stress Echocardiogram in the Era of CT Angiography

**DOI:** 10.7759/cureus.39501

**Published:** 2023-05-25

**Authors:** Kelash Kumar, Karthik Seetharam, Teesha Rani, Parvez Mir, Tanveer Mir, Vijay Shetty, Jacob Shani

**Affiliations:** 1 Internal Medicine, Maimonides Medical Center, New York, USA; 2 Internal Medicine, Wyckoff Heights Medical Center, New York, USA; 3 Medicine and Surgery, Ziauddin University, Karachi, PAK; 4 Internal Medicine and Pulmonology, Wyckoff Heights Medical Center, New York, USA; 5 Internal Medicine and Cardiology, Maimonides Medical Center, New York, USA; 6 Cardiology, Maimonides Medical Center, New York, USA

**Keywords:** cardiac ischemia, cardiac stress test, advanced cardiac imaging, computed tomography angiography (cta), stress echocardiography

## Abstract

The ideal diagnostic modality for acute chest pain is a highly debated topic in the cardiovascular community. With the rapid rise of coronary computed tomography angiography (CTA) and the fall of functional testing, stress echocardiography (SE) is at a delicate crossroads. Though there are many advantages of coronary CTA, it is not without its flaws. The exact realm of SE needs to be clearly defined, as well as which patients need diagnostic testing. The emergence of additional parameters will propel the evolution of modern SE. In this review article, we explore the role of SE, guidelines, comparison of SE versus CTA, and additional parameters in the coronary CTA era.

## Introduction and background

Acute chest pain in the emergency room can herald imminent cardiovascular conditions which require expeditious assessment and intervention [1,2]. Determining the optimal modality for acute chest pain (undifferentiated or suspected cardiac pain without ongoing ischemia) in triage is a frequently debated topic of importance [3], as non-invasive cardiac testing is the primary diagnostic consideration for these disorders [4]. The advent of computed tomography angiography (CTA) has caused paradigm shifts in non-invasive cardiac testing [3-5]. The coronary CTA is used less frequently than other functional stress testing modalities, although it is becoming more popular in the United States, whereas other functional stress testing modalities are declining. The particular benefits of coronary CTA, such as diagnostic speed [6] and strong negative predictive value, have led to broad acceptance in many institutions. As a result, there has been a widespread decrease in functional stress testing such as stress echocardiography (SE) [7].

Previously, in many earlier chest pain evaluation pathways, serial electrocardiography and cardiac biomarkers were accompanied by stress testing to rule out myocardial ischemia [8]. With the growing rise of coronary CTA, there is a need, not a necessity to evaluate the role of SE in this current era of non-invasive diagnostic testing. It is imperative to reassess the indications and criteria for SE for suspected coronary artery disease (CAD) as we embrace CTA. Furthermore, the potential significance of additional parameters and tests which can supplement SE is not known in present algorithms. In our current review, we aim to delineate the proper role of modern SE for everyday practice in CAD. 

## Review

The emergence of coronary CTA

Coronary CTA is a non-invasive diagnostic modality that permits visualization of the atherosclerotic process in CAD [9]. It is a promising diagnostic modality with several benefits. There are distinct advantages to coronary CTA over conventional modalities, which include rapid diagnostic capability [5], detection of high-risk disease, subclinical atherosclerosis, and occlusive CAD [10,11]. These supposed advantages may greatly improve the quality of patient outcomes. As mentioned earlier, CTA has a high negative predictive value [8], reduced length of stay (LOS), and a decreased time to diagnosis [5] compared to other modalities. Furthermore, this can reduce medical costs [8]. Studies have shown that CTA-based care can be financially cheaper than provocative stress testing and imaging [8]. Coronary CTA is also considered an alternative strategy to invasive coronary angiography in certain groups [2]. As per the European Society of Cardiology and the American College of Cardiology (ACC) / American Heart Association (AHA), coronary CTA can be used to exclude low to intermediate probability acute coronary syndrome cases if troponin or electrocardiogram are not considered diagnostic [12,13].

Though CTA presents a number of advantages, it is not without weaknesses [5]. One of the primary issues with coronary CTA includes elevated radiation exposure [14,15]. In addition, it can lead to further noninvasive testing [16], which is a direct result of the nature of intermediate-severity stenosis. As a result, it can lead to downstream clinical utilization [16], which can place a burden on the healthcare system. Coronary CTA has the potential to overdiagnose due to the discovery of incidental findings [5,17].

As compared to SE, the use of coronary CTA is associated with higher rates of revascularization in patients with acute chest pain. [3,18]. The exact benefit is still very unclear [3]. Though coronary CTA may have higher accuracy in identifying CAD, the extent of atherosclerosis severity may not match the physiologic lesion characteristics [19]. This technique has garnered much attention and is frequently used in comparative effectiveness research [3].

Current mindset for CAD

One of the fundamental flaws in our cardiology community is our view on revascularization [20]. Coronary revascularization is usually considered the primary treatment approach for CAD. There is a substantial disregard for medical therapy [21]. This doctrine has a domino effect on imaging and may partially be responsible for the rise of coronary CTA and the fall of stress testing [20]. Coronary CTA is frequently used as a screening tool to identify obstructive disease in asymptomatic and mildly symptomatic patients [20]. The mere presence of blockage in these patients automatically leads to revascularization. The exact impact on outcomes is not clear [3]. Low-risk CAD patients should not be advised to undergo any form of stress testing in this instance. 

The advantages of stress echocardiography

Stress echocardiography is a well-established method for detecting CAD [22]. In SE, CAD can be detected using either a diagnostic arm that includes treadmill exercise or pharmacologic stress agents such as dobutamine [23]. There has been a reported sensitivity of nearly 80% and a specificity of 85% to 90% [22]. A negative stress echocardiogram has an excellent prognosis, with a mortality rate of less than one percent per year [24]. Furthermore, in patients with suspected CAD, early stress testing plays an important role in risk stratification and rapid diagnosis. Stress echocardiography has a very low risk of ionizing radiation and is considered very safe [5].

Stress echocardiography has the distinct advantage of demonstrating the severity of physiologic lesions [10]. Furthermore, SE can link the severity of a physiologic lesion to symptoms found in a patient [10]. Once myocardial necrosis is ruled out, SE is ideal for risk management due to its high sensitivity and specificity [23]. Because of its risk stratification capabilities, SE enables rapid discharge. In addition, SE refers eligible patients to invasive coronary angiography (ICA) on an intent-to-treat basis [25].

Off note, SE is contra-indicated or not considered safe in patients with EKG findings of acute ischemia or infarction (ST-segment elevation >1mm in 2 leads or significant ST-segment depression >1mm in 2 leads), a rise in cardiac troponin (troponin-I >0.03 or troponin-T >0.1), patients with known CAD awaiting revascularization, patients in whom a non-cardiac source of chest pain is identified, patients with acute illness, serious arrhythmia, hypertrophic cardiomyopathy, severe aortic stenosis, severe left ventricular hypertrophy, and ascending aorta dilation reaching threshold for repair [23].

Stress echocardiography and coronary CTA comparison

With the blurry boundaries between coronary CTA and SE, a few centers have performed head-to-head comparisons (Table 1). Levsky et al. showed the median emergency department (ED) length of stay (p-value <0.0001), median hospital length of stay (p=0.0002), and median radiation exposure (p-value <0.0001) were smaller for SE in relation to CTA [5]. However, the number of adverse outcomes was similar between both groups. Uretsky et al. reported a higher occurrence of ICA (11% vs. 2%, p-value =0.001) and percutaneous coronary interventions (6% vs. 0%, p-value =0.001) in coronary CTA than stress testing [18]. Nevertheless, they did not find any difference in downstream testing or hospitalizations. In a meta-analysis conducted by Gongora et al., they found no differences in all-cause mortality, myocardial infarction, or major adverse cardiovascular events between CTA and SE [3]. Similarly, Gongora et al. showed a higher frequency of ICA (RR=1.32, p=0.01) and revascularization (RR=1.77, p=0.0001) in coronary CTA.

**Table 1:** Studies comparing CTA vs. standard of care CTA: Coronary tomography angiography; MPI: Myocardial perfusion imaging; ICA: Invasive coronary angiography

Study	Year	Follow-up in months	Number of subjects	Reference standard to CTA	Study findings
Dedic et al. [1]	2016	1	490	High sensitivity troponin, stress ECG, MPI 3%	CTA had less discharge (65% vs. 59%, p-value=0.16), less outpatient testing (4% vs. 10%, p-value <0.01)
Gongora et al. [3]	2018	1-19	6285	Standard of care	No significant differences in all-cause mortality. Significantly higher rates of ICA (RR 1.32, 95% CI 1.07 to 1.63, p=0.01) and revascularization (RR 1.77, 95% CI 1.35 to 2.31, p <0.0001) in coronary CTA
Goldstein et al. [4]	2007	6	197	MPI	CTA had lower diagnostic time (3.4 vs 15 hours, p-value <0.0001 lower costs p-value <0.0001, fewer repeat re-evaluations p=0.10)
Levsky et al. [5]	2015	12	400	MPI	Long term, CTA had higher all-cause radiation was lower (24 vs. 29 mSv, p=<0.0001), higher patient satisfaction (p=0.001 )
Hoffman et al. [14]	2012	1	1000	Stress ECG	CTA had a lower mean length of stay ( p-value <0.001, higher discharge from emergency department ( 47% vs. 12%, p-value <0.001 )
Uretsky et al. [18]	2016	12	395	Stress echo, MPI	CTA had a higher frequency of invasive angiography ( 11% vs. 2%, p-value <0.001 ), percutaneous coronary intervention ( 6% vs. 0%, p-value <0.001 )

How do we define the role of stress echocardiography?

Over eight million patients present to the ED in the United States with chest pain, with the possibility of acute coronary syndrome [9]. In reality, only a small percentage of patients experience CAD symptoms [9]. If coronary CTA is superior, it begs the question of how we should use SE. The European Society of Cardiology (ESC) strongly opposes stress testing in CAD patients with less than a 15% pre-test probability [26]. If a patient has a low pre-test probability, no diagnostic testing is recommended [20]. This aids in the conservation of medical resources. Patients with a pre-test probability of more than 15% should be tested.

The evolution of SE into modern SE

Wall motion assessment is the most important biomarker and the gold standard in SE [20]. Despite its high specificity, the marker has low sensitivity [27]. Nonetheless, we have a plethora of parameters at our disposal that can assist us in transitioning SE to a more modern perspective. These parameters can significantly improve SE's capabilities and sensitivity. This includes the ultrasound calcium score, global longitudinal strain in the left ventricle, Doppler coronary flow reserve (CFR), elastance for contractile reserve (CR), and contrast myocardial perfusion. Some are further along in their evolution than others [28].

Doppler coronary flow reserve on the left anterior descending

One of the important functions of SE is to optimize patient management [5]. The combination of wall motion and CFR can have a complementary effect on CAD assessment [20]. The CFR can greatly increase the prognostic value of SE and is considered favorable by the European Society of Echocardiography [29]. This combination can slightly increase the sensitivity of wall motion in SE while also slightly decreasing the specificity.

Previous research has demonstrated that abnormal CFR has a negative impact on outcomes in patients with CAD, suspected CAD, diabetics, hypertensives, and negative stress echo by wall motion criteria [20]. It is an additional parameter that can be used in the risk stratification of stress echocardiography. Patients with normal CFR can have a favorable response. Cortigiani et al. showed coronary flow reserve (hazard ratio (HR)=4.6, p-value <0.0001) and peak wall motion (HR=4, p-value <0.0001) were prognostic indicators in hypertensive patients for adverse complications, as well as in normotensive patients [30]. In another report, Cortigiani et al. showed wall motion abnormality (HR=2.43, p-value <0.0001) and ischemia in SE (HR=1.71, p-value <0.0001) were strong predictors of mortality in diabetic and non-diabetic patients [31].

Ultrasound calcium scoring

There is limited evidence that ultrasound calcium in conjunction with SE has cardiovascular prognostic value. By echocardiography, it is well established that calcification of the aortic or mitral valve is associated with cardiovascular morbidity and mortality [32,33]. The echocardiographic calcium score (eCS) can supplement the SE-derived wall motion values. Unlike other ancillary tests, it does not necessitate highly advanced machines and is relatively inexpensive, making it appealing for acceptance to support SE [20]. Gaibazzi et al. discovered that ischemic SE or eCS greater than zero was associated with a poor prognosis (p-value <0.0001) and contributed more to risk prediction than clinical factors [34]. Gabazzi et al. previously demonstrated that a calcification score index (CSI) greater than zero was the strongest independent predictor of SE ischemia in comparison to other clinical factors (OR=2.15 (1.48-3.13), (p-value <0.0001) [35].

Global strain assessment during stress echocardiography

Though strain is gaining momentum for providing information regarding myocardial function beyond ejection fraction, it is still in the early stages. The strain provides valuable information regarding cardiac deformation; it measures the transition from a contractile state to a relaxed state [36]. Longitudinal strain imaging during SE has better diagnostic accuracy for detecting significant CAD as compared to visual assessment of wall motion abnormality (WMA). The incremental value of strain in combination with wall motion assessment has been explored in relatively few studies [37-39]. Further investigation is needed to clinically understand its full potential. Ng, et al. showed the combination of longitudinal strain and wall motion assessment had 100 percent sensitivity and 87.5 percent specificity, and the diagnostic accuracy was incremental to strain (p=0.0034) or wall motion assessment (p=0.008) [40]. Piccione et al. showed that global longitudinal strain improved the accuracy of SE for single vessel CAD in comparison to wall assessment alone for peak and low doses [41]. Gabazzi et al. showed stress WMAs (p-value <0.0001) and rest longitudinal global strain (p-value <0.0001) were significant predictors of CAD, and strain helped improve the accuracy of stress echo data for obstructive disease [38].

Elastance reserve is also known as contractile reserve

Elastance reserve has the potential to be a valuable diagnostic and prognostic tool. It may add value to wall motion measurements obtained with SE [42]. It does not necessitate any kind of learning curve. The technique is based on the relationship between end-systolic pressure and volume [42]. There have been a few studies with the aforementioned techniques. Elastance reserve was found to increase the sensitivity of wall motion assessment to 88% while decreasing the specificity to 83% in patients with positive stress (-3.6 SD, 3 mmHg/ml/m2) [42]. Cortigiani et al. discovered that CR (HR=2.28, p=0.03) and resting WMA (HR = 1.94, p-value = 0.02) were predictors of future events in diabetics with negative SE; elastance reserve added an additional layer of prognostic stratification to regional wall motion assessment [31].

Contrast myocardial perfusion

Among the different parameters mentioned, contrast myocardial perfusion has been hailed as a promising diagnostic approach [20]. Additional guidelines may be required for successful clinical practice implementation. When two or more continuous segments are not visible at rest on SE, the European Society of Cardiovascular Imaging (ESCVI) recommends using contrast [43]. If the left ventricular wall motion is assessed with contrast agents, it can be administered if there are less than two segments of visualization. It is critical to note that for SE, only low-contrast myocardial perfusion should be used [43].

Despite its many advantages, contrast echocardiography is not widely used. In comparison to other parameters, it is not as simple to use. Furthermore, the FDA has not approved many contrast agents for use in perfusion imaging [43]. Few studies have fully explored the potential of contrast myocardial perfusion imaging in conjunction with myocardial stress assessment (Table 2). With the addition of contrast myocardial perfusion imaging, Gaibazzi et al. demonstrated higher sensitivity of wall motion assessment in isolated intermediate stenosis at 98% (p-value <0.0001) but lower incremental sensitivity in severe stenosis at 96% (p-value <0.05) [44]. In another study, Gaibazzi et al. found that combining contrast myocardial perfusion imaging with SE could identify 71 true positive findings of cardiac chest pain compared to 46 by sole wall motion assessment (p-value <0.05), with a non-significant difference in positive predictive value [45]. Elhendy et al. found that myocardial contrast perfusion imaging had higher sensitivity than wall motion assessment at maximal stress (91% vs. 70%, p=0.0001) and intermediate stress (84% vs. 20%, p=0.0001), but lower specificity (p=0.01) in CAD patients [46].

**Table 2:** Examples of additional parameters added to wall motion assessment CFR: Coronary flow reserve; LAD: Left anterior descending; ROC: Receiver-operating characteristics; CI: Confidence interval; OR: Odds ratio; GLS: Global longitudinal strain; CAD: Coronary artery disease; WM: Wall motion; WMA: Wall motion abnormality; MPI: Myocardial perfusion imaging; CR: Contractile reserve; HR: Hazard ratio

Study	Year	Study size	Additional parameter	Study findings
Gaibazzi et al. [28]	2010	400	Doppler coronary flow reserve	Combined wall motion assessment and CFR-LAD obtained intermediate values for both sensitivity (84%) and specificity (71%) and second-best accuracy (80%)
Cortigiani et al. [30]	2011	2089	Doppler coronary flow reserve	The CFR had the best ROC for diagnosis of LAD stenosis in hypertensive (0.86) and normotensive (0.90). The CFR was a predictor for age in hypertensive (HR=3.1) and normotensive (HR=3.4)
Cortigiani et al. [31]	2009	233	Contractile reserve	Lower event-free survival with peak contractile reserve ≤28 (p=0.006), CI: 1.08 to 4.81 was a predictor of future events (HR=2.28, p=0.03)
Gaibazzi et al. [34]	2015	1303	Ultrasound calcium scoring	Calcium score >0 was a predictor of hard events, worse outcomes on Kaplan-Meier. When SE and calcium score were abnormal, it had a worse prognosis (p-value <0.001)
Gaibazzi et al. [35]	2014	1117	Ultrasound calcium scoring	Calcium score was an independent predictor of ischemia at SE (OR 2.15), added significant incremental value
Ng et al. [40]	2009	102	Myocardial strain	Longitudinal strain had comparable accuracy to the wall motion stress index (p=0.70). The combination of longitudinal strain and wall motion index had the highest sensitivity, specificity, and accuracy (100%, 87.5%, and 96.3%)
Cusma-Piccione et al. [41]	2015	52	Myocardial strain	GLS had higher sensitivity (61%) and specificity (90%) than wall motion assessment with 44% sensitivity and 55% specificity in CAD detection
Bombardini et al. [42]	2013	111	Contractile reserve	The sensitivity of WMA, CFR, and CR had 88% sensitivity for CAD. High prevalence of impaired CR with WMA-detected CAD
Gaibazzi et al. [45]	2009	400	Contrast myocardial perfusion	WM and MPI resulted in 71 true positive findings compared to 46 with WM only (p-value <0.05)
Elhendy et al. [46]	2004	170	Contrast myocardial perfusion	The sensitivity of myocardial contrast echocardiography was higher than WMA at maximal stress (91% vs. 70%, p= 0.0001) and intermediate stress (84% vs 20%, p=0.0001). Lower specificity (91% vs. 70%, p=0.0001)

The real potential significance of the ancillary tests

Though several tests are mentioned, many of them are still in the works. Many centers in the United States do not use all of these tests; instead, they focus primarily on research. Only a few of these, in reality, have the potential to be at the forefront of wall motion assessment in SE. The most promising are CFR and contrast echocardiography [20]. These tests require skill and possibly training for the novice echocardiographer to become proficient. Nonetheless, each test has advantages and disadvantages (Table 3).

**Table 3:** Advantages and disadvantages of additional parameters in stress echocardiography CFR: Coronary flow reserve; SE: Stress echocardiography

Additional parameter	Advantages	Disadvantages
Doppler CFR	Low cost, not difficult to perform	Decreases specificity of SE
Ultrasound calcium scoring	Low cost, easy to perform	Not useful if the calcium score is trace or low
Global longitudinal strain	Incremental diagnostic accuracy	Time-consuming, learning curve, requires special software
Elastance or contractile reserve	Easy to calculate and perform	Limited literature
Contrast myocardial perfusion	Good diagnostic and prognostic capability	Learning curve

Coronary flow reserve does not require any form of contrast media administration. Nonetheless, it can only be utilized for the left anterior descending artery measurement. Contrast echocardiography can provide measurements in all coronary regions. Contrast echocardiography does not necessarily improve accuracy but is more practical to use.

The rise and relevance of machine learning

In recent years, there has been a rapid rise in the complexity and size of data in healthcare systems [47]. This explosive growth has also been evidenced in cardiovascular imaging. The vast data matrix present within these massive databases cannot be tapped by conventional statistical approaches. With the advent of machine learning (ML), a subset of artificial intelligence (AI), these algorithms can unravel new relationships within these datasets, which can positively impact patient welfare. Recently, Omar et al. compared a convolutional neural network (CNN) with manual contouring for wall motion assessment. The CNN approach has 85.4% accuracy, 92.8% sensitivity, and 77.6% specificity [48].

The rate of ML growth has been highest in the field of echocardiography [47]. With the evolution of SE in this current era of CT angiography, ML algorithms can be useful for standardization of interpretation and improved efficiency despite the addition of new variables for assessment. 

## Conclusions

As we move forward in this CTA era, the role of modern SE must be clearly defined. The distinct advantage of stress testing is that it correlates lesion severity with physiological symptoms. The assessment of wall motion in SE is a gold standard with high specificity and low sensitivity. Sensitivity can be greatly increased by using additional parameters such as CFR and myocardial contrast perfusion imaging. Finally, with the addition of variables, the rise of ML will aid in the standardization of interpretation and improve efficiency.
